# Neuroprotective effects of pifithrin-α against traumatic brain injury in the striatum through suppression of neuroinflammation, oxidative stress, autophagy, and apoptosis

**DOI:** 10.1038/s41598-018-19654-x

**Published:** 2018-02-05

**Authors:** Ya-Ni Huang, Ling-Yu Yang, Nigel H. Greig, Yu-Chio Wang, Chien-Cheng Lai, Jia-Yi Wang

**Affiliations:** 1 Department of Nursing and Department of Optometry, Hsin Sheng Junior College of Medical Care and Management, Taoyuan City, Taiwan; 2 Graduate Institute of Medical Sciences, College of Medicine, Taipei Medical University, Taipei, Taiwan; 3 Drug Design & Development Section, Translational Gerontology Branch, Intramural Research Program, National Institute on Aging, National Institutes of Health, Baltimore, MD USA; 4 School of Medicine, College of Medicine, Taipei Medical University, Taipei, Taiwan; 5 Division of Orthopedics, Department of Surgery, Far Eastern Memorial Hospital, New Taipei City, Taiwan; 6 Department of Physiology, College of Medicine, Taipei Medical University, Taipei, Taiwan

## Abstract

Cortical and hippocampal neuronal damages caused by traumatic brain injury (TBI) are associated with motor and cognitive impairments; however, only little attention paid to the striatal damage. It is known that the p53 tumor-suppressor transcription factor participated in TBI-induced secondary brain damage. We investigated how the p53 inactivator pifithrin (PFT)-α affected TBI-induced striatal neuronal damage at 24 h post-injury. Sprague-Dawley rats subjected to a controlled cortical impact were used as TBI models. We observed that p53 mRNA significantly increased, whereas p53 protein expression was distributed predominantly in neurons but not in glia cells in striatum after TBI. PFT-α improved motor deficit following TBI. PFT-α suppressed TBI-induced striatal glial activation and expression of proinflammatory cytokines. PFT-α alleviated TBI-induced oxidative damage TBI induced autophagy was evidenced by increased protein expression of Beclin-1 and shift of microtubule-associated light chain (LC)3-I to LC3-II, and decreased p62. These effects were reduced by PFT-α. Post-injury PFT-α treatment reduced the number of degenerating (FJC-positive) and apoptotic neurons. Our results suggest that PFT-α may provide neuroprotective effects via p53-dependent or -independent mechanisms depending on the cell type and timing after the TBI and can possibly be developed into a novel therapy to ameliorate TBI-induced neuronal damage.

## Introduction

Traumatic brain injuries (TBIs) are a serious health problem worldwide. TBIs range from mild to severe, and most victims of TBI have mild injury characterized by short- and long-term physical, emotional, behavioral, and cognitive impairments consequent to the brain damage^1^. It is becoming increasingly evident that dysregulation of the dopamine (DA) system may be a major contributing mechanism for behavioral and cognitive deficits after TBI^2–4^. Reductions in striatal tyrosine hydroxylase activity and DA release in the striatum of rats were reported after TBI^5^. TBI-induced striatal mitochondrial disruption causes behavioral deficits^6^. Those findings suggest that loss of function of the striatal DA system and/or striatal damage after TBI may be major contributing mechanisms for behavioral impairments. However, the underlying cellular and molecular mechanisms still need to be further explored.

The TBI mechanism is a complicated pathological process that consists of a primary insult (mechanical impact) and secondary (delayed) insults. Hallmarks of the secondary insult response in TBIs include generation of neuroinflammation, reactive oxygen species (ROS), autophagy and apoptotic signaling cascades, all of which occur immediately following the primary mechanical insult^1,7–9^. Evidence strongly suggests that the generation of ROS is an early event following brain injury, that occurs within minutes of a mechanical impact^9,10^. When cellular damage is sufficiently profound, proapoptotic proteins (e.g., Bax and cleaved caspase-3) and transcription factors (nuclear factor (NF)-κB and p53) initiate the process of neuronal cell death. p53, a tumor-suppressor gene, is critical for activating expression of genes engaged in promoting cell-cycle arrest or cell death in response to various cellular stresses^11^. p53’s activity is controlled by transcriptional, translational, and post-translational regulatory mechanisms. In response to cellular stress, p53 induces its biological response through the transcriptional transactivation of specific target genes such as the proapoptotic genes, Bax and PUMA^12^. Inappropriate p53 activation is involved in stress-induced apoptotic signaling pathways in neuronal diseases and brain injuries. It was demonstrated that p53-induced apoptosis plays a crucial role in the development of hypoxic-ischemic brain damage^13^. Inhibition of the p53 pathway improves functional outcomes and attenuates lesion volumes after hypoxia-ischemia in the rat brain^14^. Accumulating evidence also indicates a primary role of p53 in neuronal death that occurs following TBI^15–17^. Activation of c-Jun N-terminal kinase (JNK) signaling was shown to mediate neuronal cell damage after TBI^18,19^. It is known that p53 is a downstream JNK signaling. The damage-regulated autophagy modulator (DRAM), a p53 target gene, is involved in autophagy formation^18,20^. DRAM is involved in regulating nucleoside analog-induced neuronal autophagy in a p53-independent manner^21^. However, p53 phosphorylation after a TBI enhances neuron autophagy through upregulating DRAM and disruption of Beclin-1 complex p53 signaling in neuronal apoptosis and autophagy formation as reported by Hong *et al*.^18^, also suggesting a p53-dependent pathway.

Autophagy, a lysosomal pathway for degrading and recycling macromolecules and organelles, plays an essential role in differentiation, development, and cellular homeostatic functions^22,23^. ROS are essential for inducing autophagy formation^22^. In the absence of tight regulation, autophagy accelerates the apoptotic destruction of cells via self-digestion, thus leading to cell damage. Autophagy is involved in type II (autophagic) cell death, and is distinguished from type I (apoptotic) cell death^24^. The appearance of autophagic cell death is characterized by the accumulation of autophagic vesicles in dying cells^24^. Autophagy is negatively regulated by mammalian target of rapamycin (mTOR) and its downstream cascades of autophagy-related proteins (Atg), such as Beclin-1 and microtubule-associated light chain 3 (LC3)^23,25^. The mammalian homologue of Atg6, also called Beclin-1, is essential for initiating autophagy via its interaction with class III phosphatidylinositol-3 (PI3) kinase^26^. Autophagic flux Antiapoptotic proteins (e.g., Bcl_2_ and Bcl_XL_) can bind to Beclin-1 and reduce the PI3-kinase activity associated with Beclin-1, thus inhibiting autophagy formation^27^. In a murine encephalitis model, Beclin-1 interacted with Bcl_2_ to protect neuronal cells against virus-induced cell death^28^. Dissociation of the Bcl_2_/Beclin-1 complex was observed in methamphetamine-induced autophagy, thus leading to cell death^29^. Those findings suggest that interactions between antiapoptotic and autophagic proteins play important roles in cytoprotection and/or cell death.

Pifithrin (PFT)-α, a p53 inhibitor, was found to prevent neuronal cell death by inhibiting p53 transcriptional activity, mitochondrial damage, and caspase activation^30,31^. PFT-α was demonstrated to be effective in preventing neuronal cell death in models of ischemia, excitotoxic insults, and Parkinson’s disease^32–34^. Those results indicated that inhibition of p53 by PFT-α may prove to be a useful tool to evaluate p53-dependent cascades and may also be developed into a novel therapeutic strategy for TBIs. In the present study, we focused on effects of PFT-α in neuroinflammation, oxidative stress, autophagy, and apoptotic cell death in rats following experimental administration of TBI.

## Results

### Post-injury PFT-α treatment inhibits the transcriptional and translational expression of p53 in neurons at 24 h after TBI

**Figure 1:**
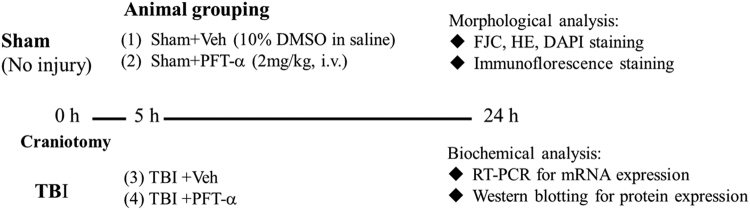
Experimental design. Anesthetized male SD rats were subjected to either TBI with a control cortical impactor (CCI) at a velocity of 4 m/s and a deformation depth of 2 mm below the dura) or a sham procedure (craniotomy without CCI) and an iv. injection of either pifithrin (PFT)-α (2 mg/kg) or a vehicle (Veh; 10% DMSO in saline) were administered 5 h later. Animals were sacrificed at 24 h. Tissue sections were fixed and sectioned for morphological analyses (e.g., H&E, DAPI, FJC and immunofluorescence staining) and striatal tissues were isolated for biochemical analyses (measurement of mRNA and protein levels).

**Figure 2:**
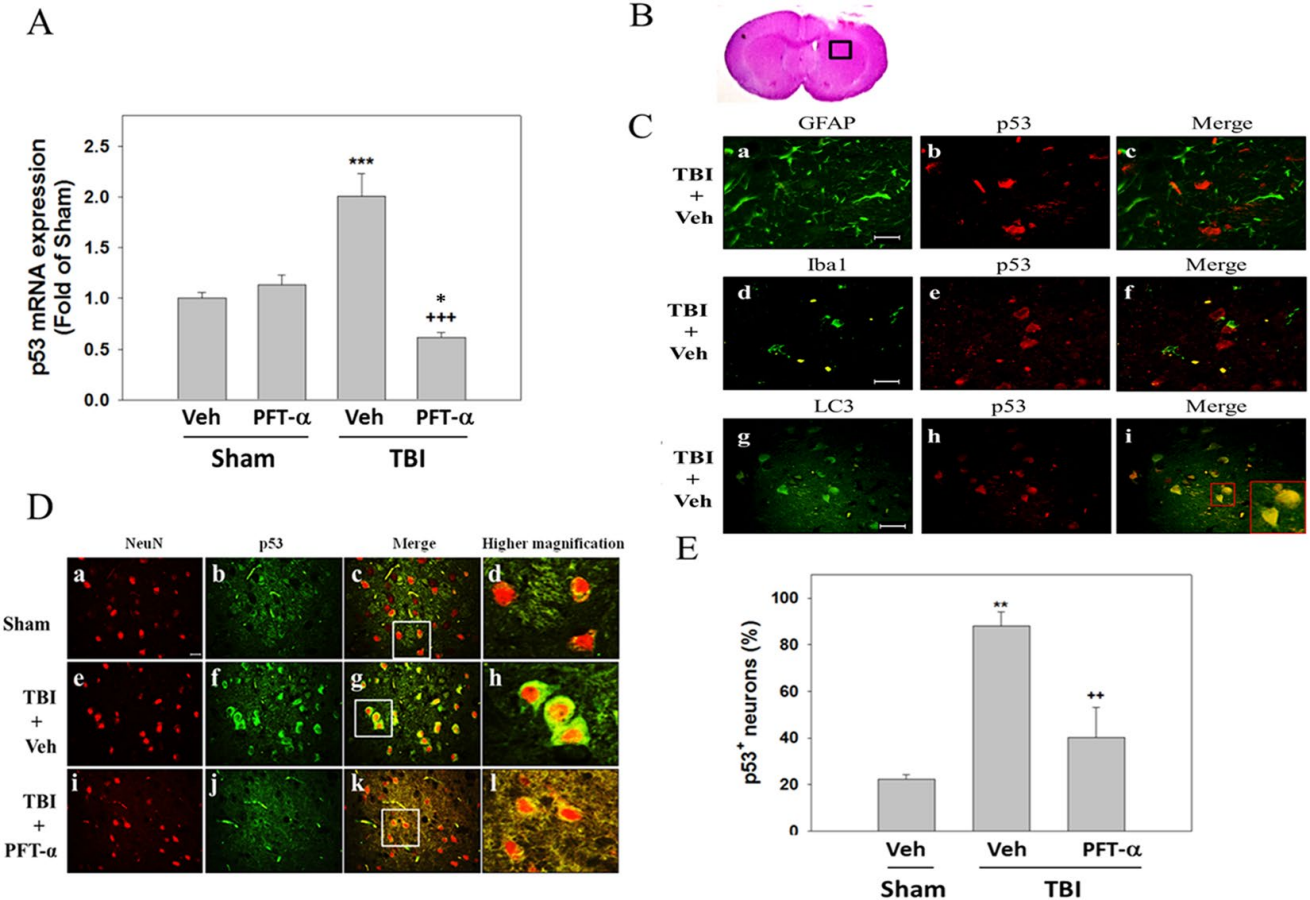
Post-injury administration of pifithrin (PFT)-α at 5 h after a traumatic brain injury (TBI) reduced levels of p53 mRNA expression and the number of p53-positive neurons in striatal tissues at 24 h. (**A**) mRNA levels of p53 in striatal tissues from the Sham + vehicle (Veh), Sham + PFT-α, TBI + Veh, and TBI + PFT-α groups were analyzed by an RT-PCR. Multiples of change are relative to that of the sham group. (**B**) A lower magnification H&E-stained coronal brain section from the TBI + Veh animal showing the dorsal striatum where the higher magnification images are taken. (**C**) Photomicrographs showing the results of immunofluorescence staining using anti-GFAP (a maker for astrocytes, green in a), anti-Iba1 (a maker for microglia, green in d), or LC3 (a maker for autophagy, green in g) combined with anti-p53 (red in b, e and h). Merged images show that p53 is not colocalized with GFAP (c), or Iba1 (f), but is colocalized with LC3 (i) in striatal tissues from TBI + Veh animals. Inset, a high-power image showing colocalization of p53 and LC3. Scale bar = 50 µm. (**D**) Double immunofluorescence staining of NeuN and p53 in striatal brain tissues. NeuN is shown in red, and p53 is shown in green. Yellow labeling indicates colocalization. (**E**) Quantitative comparison of NeuN-p53-positive cells in the Sham + Veh, TBI + Veh, and TBI + PFT-α groups. Data are expressed as the mean ± SD (*n* = 5 per group). *P < 0.05, **P < 0.01 and ***P < 0.001 vs. Sham + Veh group; ^++^P < 0.01 and ^+++^P < 0.001 vs. TBI + Veh group.

According to our experimental design (Fig. 1), we first examined the effects of PFT-α on p53 mRNA and protein expression in striatal tissues at 24 h after TBI respectively using RT-PCR and immunofluorescence staining. Results showed that levels of p53 mRNA were significantly elevated in the TBI-treated group (2.0 ± 0.23-fold, P < 0.001) compared to the sham-treated group (1 ± 0.06-fold); however, this effect was significantly attenuated in the PFT-α treatment group (0.61 ± 0.05-fold, P < 0.001) when PFT-α was administered 5 h following the TBI (Fig. 2A). Levels of p53 expression in the Sham + PFT-α group (1.1 ± 0.1-fold) did not significantly differ from those observed in the Sham + Veh group (1 ± 0.06-fold). Results show that levels of p53 mRNA expression in TBI + PFT-α group were significantly lower than those observed in Sham + Veh group (0.61 ± 0.05-fold vs. 1 ± 0.06-fold, P < 0.05) but were not significantly different from Sham + PFT-α group (P = 0.053). Double immunofluorescence staining was carried out on coronal sections of the dorsal striatum (Fig. 2B) from TBI + Veh animals using an antibody against p53 combined with either GFAP (a marker of astrocytes) or Iba1 (a marker of microglia). Results showed that TBI-induced p53 expression occurred in neither GFAP-positive astrocytes (Fig. 2Ca–c) nor Iba1-positive microglia (Fig. 2Cd–f) in the striatum. Interestingly, TBI-induced p53 expression was colocalized with microtubule-associated light chain 3 (LC3), suggesting a possible correlation with autophagy (Fig. 2Cg–i). Double immunofluorescence staining using anti-p53 combined with a NeuN antibody (a marker for neurons) showed a few p53-positive neurons in the sham group (Fig. 2Da–d). However, more p53-positive neurons in the TBI + Veh group (Fig. 2De–h). Post-injury treatment with PFT-α at 5 h significantly reduced the number of p53-positive neurons (Fig. 2Di–l). A quantitative analysis revealed that the number of p53-positive neurons significantly increased at 24 h in the TBI + Veh group after injury compared to the Sham + Veh group (88.2 ± 5.9% vs. 22.3 ± 1.8%, P < 0.01). PFT-α administration reduced the number of p53-positive neurons compared to the TBI-treated group(40.2 ± 12.9% vs. 88.2 ± 5.9%, P < 0.01) (Fig. 2E).

### Post-injury treatment with PFT-α at 5 h improved neurological functional deficits after TBI

**Figure 3:**
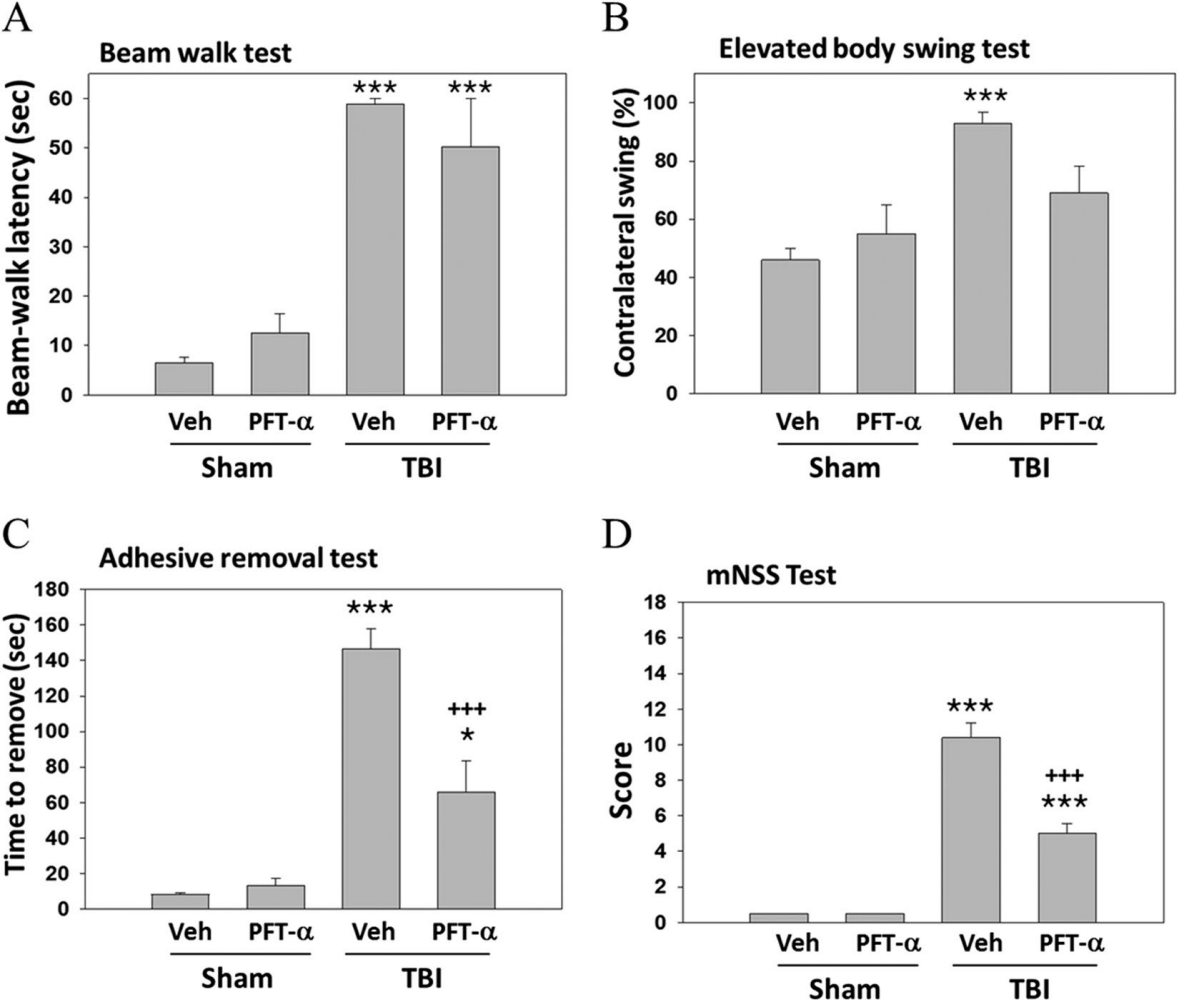
Post-injury administration of PFT-α at 5 h improved neurological functional outcomes as revealed by behavioral evaluation. (**A**) Motor coordination measured by beam walk test. (**B**) Motor asymmetry measured by elevated body swing test (EBST). (**C**) Sensory-motor function measured by adhesive removal test. (**D**)Neurological function measured by mNSS. Data represent the mean ± SEM (n = 4 per group). *P < 0.05 and ***P < 0.01 vs. Sham + Veh group; ^+++^P < 0.001 vs. TBI + Veh group.

Baseline values in all tests did not significantly differ in the Sham + Veh, Sham + PFT-α, TBI + Veh, and TBI + PFT-α groups (data not shown). Motor coordination impairment, measured by a beam walking test, was evident after a TBI in vehicle-treated animals at 24 h (Fig. 3A); however, post-injury treatment with PFT-α caused no differences in the beam walking test compared to the TBI + Veh group. The functional evaluation measured by the elevated body swing test (EBST) at 24 h after a TBI in sham and TBI rats indicated that impaired functional outcomes, as measured by motor asymmetry, caused by the TBI were evident in the vehicle-treated group (Fig. 3B). Post-injury treatment with PFT-α slightly but not significantly improved functional deficits after the TBI, as revealed by swing tests (Fig. 3B). Functional evaluation measured by tactile adhesive removal at 24 h after the TBI in sham and TBI rats indicated that functional deficits caused by the TBI were evident in the vehicle-treated group (Fig. 3C), in which a unilateral TBI resulted in a delay in the time needed to remove the patch. Post-injury treatment with PFT-α improved the functional deficits after the TBI, as revealed by the tactile adhesive removal test (Fig. 3C, P < 0.01). In addition, neurological function as measured by mNSS scores at 24 h after the TBI in sham and TBI rats indicated that the overall functional outcome caused by the TBI was impaired in the vehicle-treated group. Post-injury treatment with PFT-α significantly improved functional deficits after the TBI, as revealed by mNSS scores (Fig. 3D; P < 0.001). All tests in Sham + PFT-α animals revealed no significant difference from those observed in Sham + Veh animals.

### Post-injury PFT-α treatment inhibits glial cell activation

**Figure 4:**
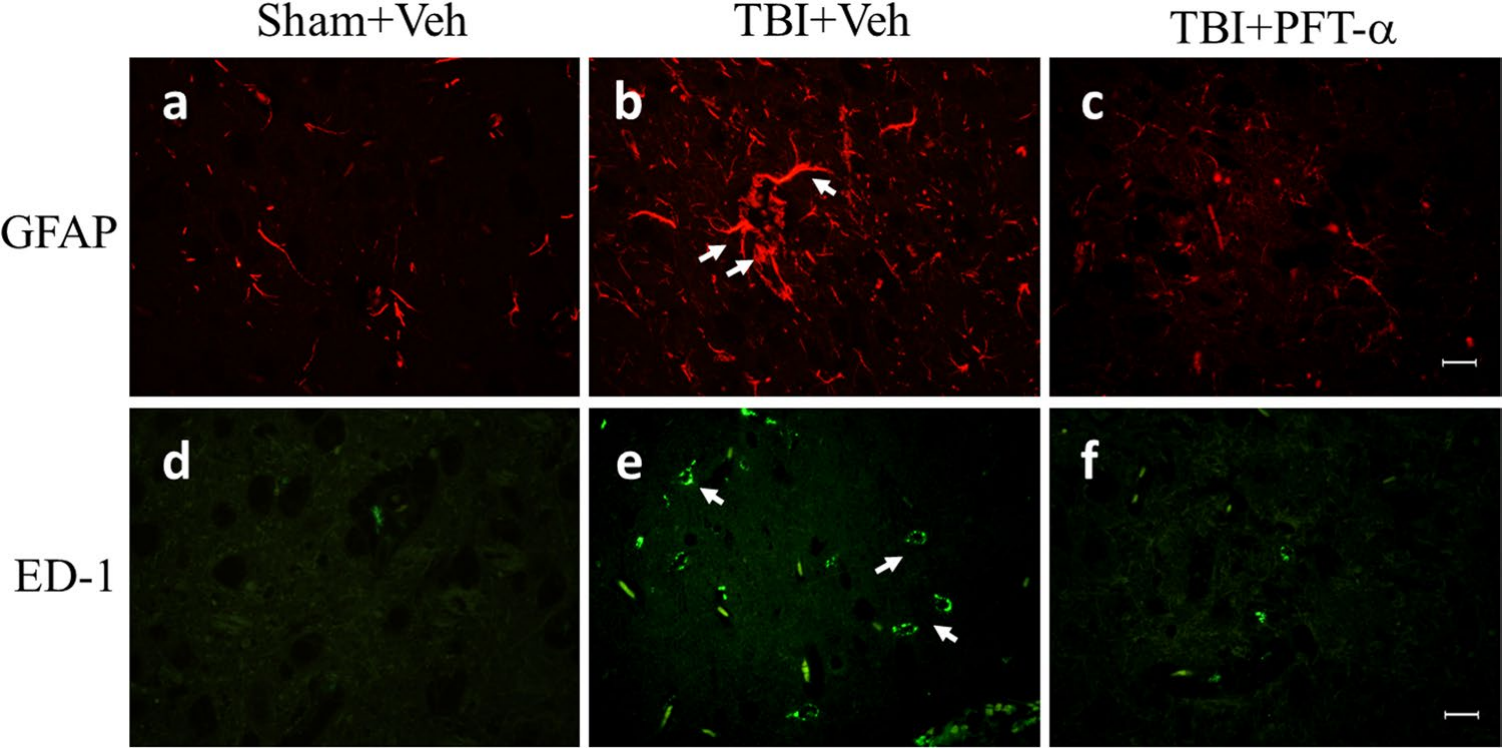
Post-injury administration of pifithrin (PFT)-α at 5 h after a traumatic brain injury (TBI) alleviated glial activation in the striatum of TBI rats. Representative photomicrographs show brain sections from the Sham + Veh (**a**,**d**), TBI + Veh (**b**,**e**), and TBI + PFT-α (**c**,**f**) groups stained with antibodies to identify astrocytes (GFAP-positive cells; **a**–**c**) and microglia (ED1-positive cells; **d**–**f**).

IHC staining showed that astrocytes that exhibited GFAP immunoreactivity displayed a polygonal profile, with filament-like immunoreactivity in the cytoplasm of the sham group (Fig. 4a) vs. stellate soma and hypertrophic processes, typical morphological characteristics of activated astrocytes, in the TBI + Veh group (Fig. 4b). Expression of activated microglia was negligible in the Sham + Veh group (Fig. 4d), whereas ED-1-positive cells from the TBI + Veh group had an amoeboid morphology that is characteristic of reactive microglia (Fig. 4e). However, both astrocyte and microglia activation was attenuated by PFT-α treatment (Fig. 4c,f, respectively).

### Post-injury PFT-α treatment reduces transcriptional and translational levels of TNF-α, IL-1β, and IL-6

**Figure 5:**
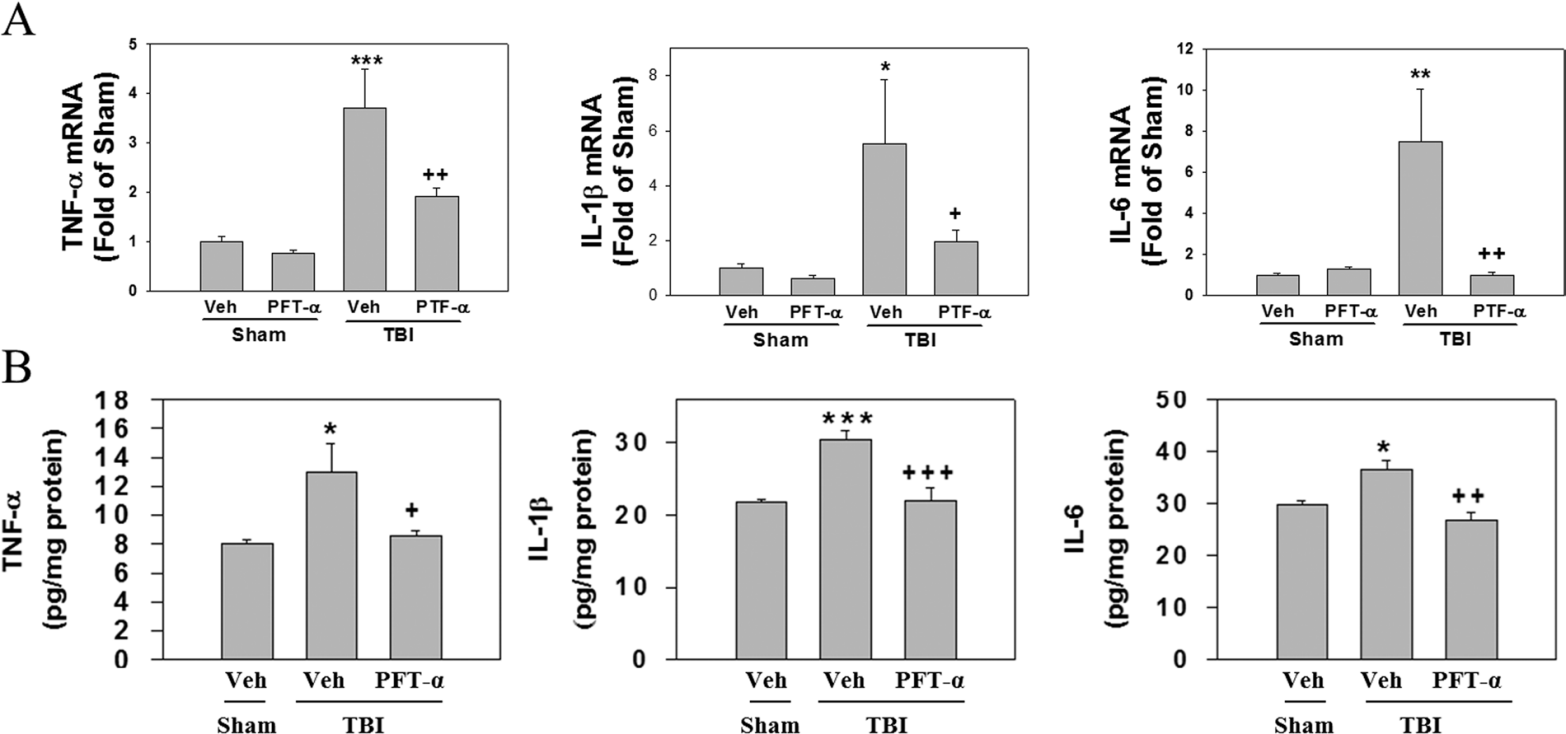
Post-injury administration of pifithrin (PFT)-α at 5 h after a traumatic brain injury (TBI) reduced the proinflammatory cytokines tumor necrosis factor (TNF)-α, interleukin (IL)-1β, and IL-6 levels in striatal tissues at 24 h. (**A**) mRNA levels of TNF-α, IL-1β, and IL-6 in brain tissues from Sham + Veh, Sham + PFT-α, TBI + Veh, and TBI + PFT-α groups were analyzed by an RT-PCR. Multiples of change are relative to that of the Sham + Veh group. (**B**) Levels of TNF-α, IL-1β, and IL-6 in brain tissues from Sham + Veh, Sham + PFT-α, TBI + Veh, and TBI + PFT-α groups were measured by an ELISA. Data are expressed as the mean ± SD (*n* = 5 per group). *P < 0.05, **P < 0.01, and ***P < 0.001 vs. Shan + Veh group; ^+^P < 0.05, ^++^P < 0.01, and ^+++^P < 0.001 vs. TBI + Veh group.

We examined the effects of PFT-α on TNF-α, IL-1β, and IL-6 mRNA and protein expression in striatal tissues at 24 h after TBI respectively using RT-PCR and ELISA assays. Results showed that levels of TNF-α (3.71 ± 0.78-fold, P < 0.001), IL-1β (5.53 ± 2.31-fold, P < 0.05), and IL-6 (7.5 ± 2.53-fold, P < 0.01) mRNAs were significantly elevated in the TBI-treated group compared to the sham-treated group (1 ± 0.1-fold, 1 ± 0.14-fold, and 1 ± 0.1-fold, respectively); however, this effect was significantly attenuated in the PFT-α treatment group (1.91 ± 0.16-fold, P < 0.01; 1.96 ± 0.42-fold, P < 0.05; and 1 ± 0.14-fold, P < 0.01, respectively) when administered 5 h following the TBI (Fig. 5A). Levels of TNF-α, IL-1β, and IL-6 mRNAs in the Sham + PFT-α group (0.76 ± 0.07-fold, 0.63 ± 0.09-fold, and 1.28 ± 0.1-fold, respectively) did not significantly differ from those observed in the sham group (Fig. 5A). Regarding protein expression, levels of TNF-α (13 ± 0.1.9 pg/mg protein, P < 0.05), IL-1β (30.48 ± 1.27 pg/mg protein, P < 0.001), and IL-6 (36.56 ± 1.56 pg/mg protein, P < 0.05) protein had significantly increased in the TBI-treated group, whereas these effects were significantly reduced by PFT-α treatment (8.52 ± 0.39 pg/mg protein, P < 0.05; 21.96 ± 1.81 pg/mg protein, P < 0.001; and 26.67 ± 1.55 pg/mg protein, P < 0.01, respectively) (Fig. 5B).

### Post-injury PFT-α treatment induces HO-1 expression and attenuates lipid peroxidation (LP) in the striatum of rats with TBI

**Figure 6:**
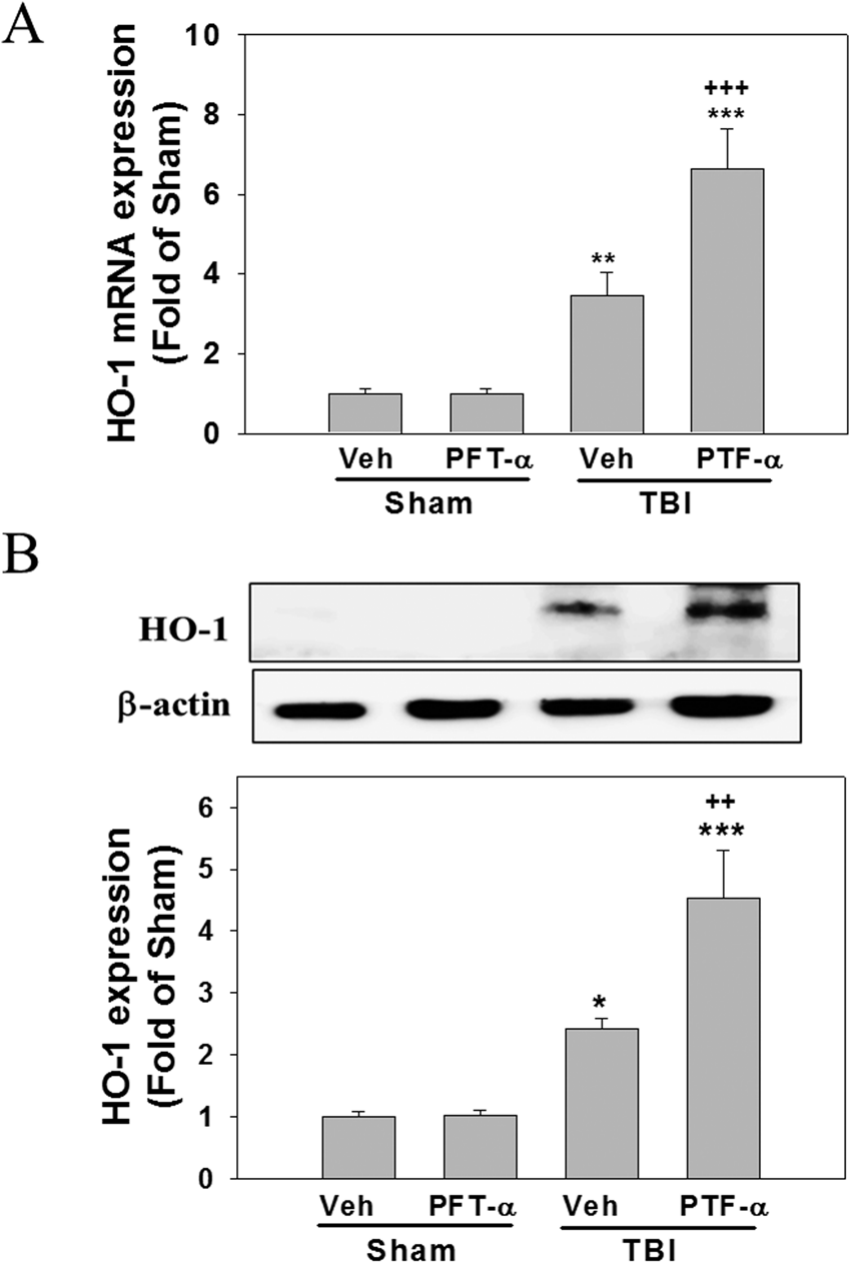
Post-injury administration of pifithrin (PFT)-α at 5 h after a traumatic brain injury (TBI) enhanced levels of heme oxygenase (HO)-1 expression in striatal tissues at 24 h. Levels of HO-1 expression in striatal tissues from the Sham + Veh, Sham + PFT-α, TBI + Veh, and TBI + PFT-α groups were analyzed by Western blotting. Levels of HO-1 expression were normalized to β-actin and then quantified. Multiples of change are relative to that of the Sham + Veh group. Data are expressed as the mean ± SD (*n* = 3 per group). *P < 0.05 vs. Sham + Veh; ^+^P < 0.05 vs. TBI + Veh group.

**Figure 7:**
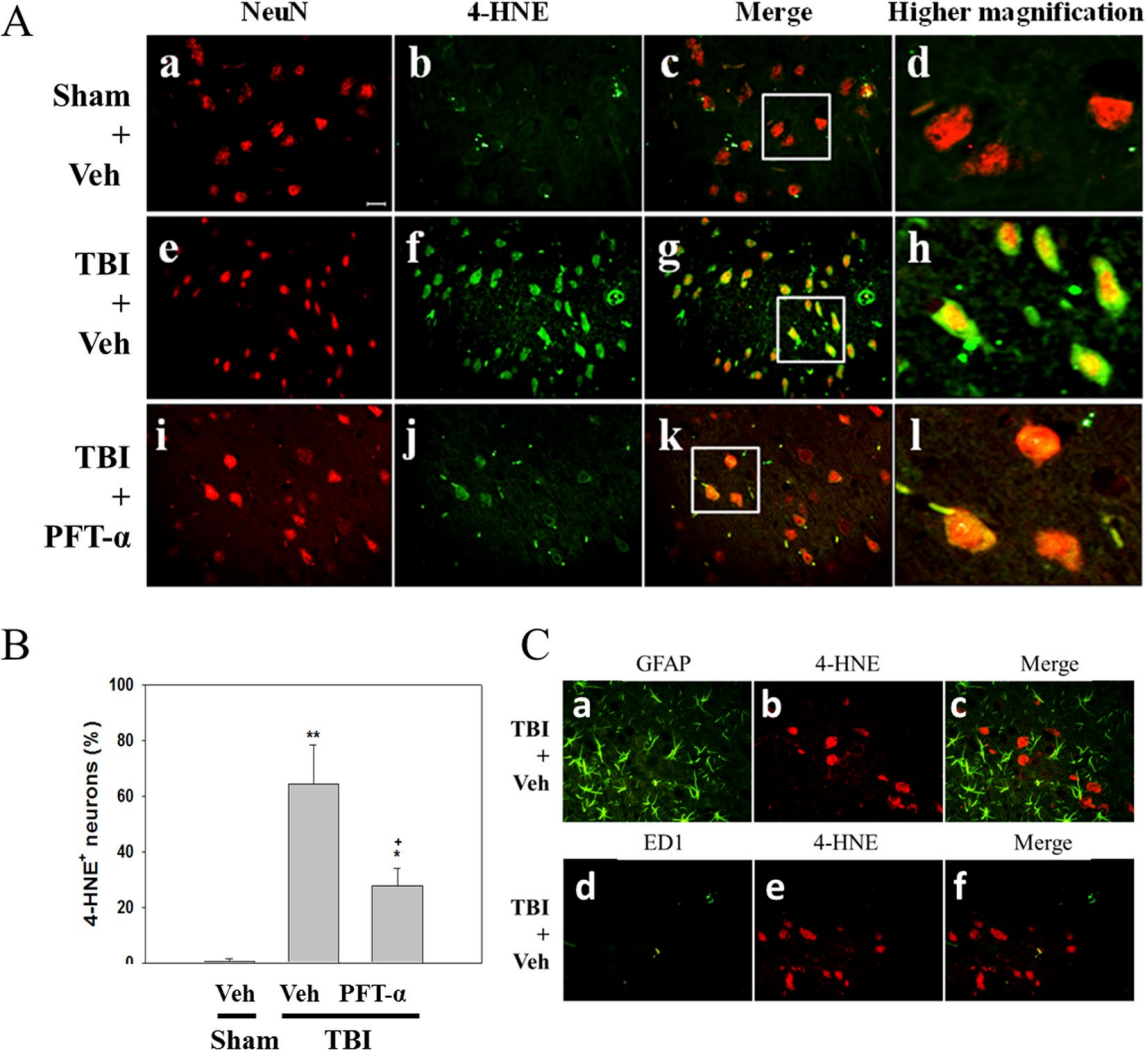
Post-injury administration of pifithrin (PFT)-α at 5 h after a traumatic brain injury (TBI) reduced 4-hydroxynonenal (HNE)-positive neurons in striatal tissues at 24 h. (**A**) Double immunofluorescence staining of NeuN and 4-HNE in striatal brain tissues. NeuN is shown in red, and 4-HNE is shown in green. Yellow labeling indicates colocalization. Scale bar = 50 μm. (**B**) Quantitative comparison of NeuN-4-HNE-positive cells in the Sham + Veh, TBI + Veh, and TBI + PFT-α groups. Data are presented as the mean ± SEM (*n* = 3 per group). *P < 0.05 and **P < 0.01 vs. Sham = Veh group; ^+^P < 0.05vs. TBI + Veh group. (**C**) Micrographs show immunofluorescence staining using anti-GFAP (green in a), anti-ED1 (green in d), or anti-4-HNE (red in b and e). Merged images show that there is no colocalization of GFAP and 4-HNE (c), or ED1and 4-HNE after TBI.

Oxidative stress is a key mediator exacerbating neuronal damage and contributing to the pathogenesis of TBIs^35–37^. HO-1 is well known as the 32-kDa heat shock protein which is upregulated by oxidative stress and appears to be a component of the cellular oxidative stress response^38–40^. Levels of HO-1 mRNA expression in the Sham + PFT-α group did not significantly differ from those observed in the Sham + Veh group (Fig. 6A). Results showed that HO-1 mRNA (Fig. 6A) and protein levels (Fig. 6B) were significantly elevated in the TBI + Veh-treated group (3.07 ± 0.95-fold and 2.24 ± 0.09-fold, respectively) compared to the Sham + Veh group (1.00 ± 0.15-fold and 1.00 ± 0.09-fold, respectively), these effects were significantly enhanced in the TBI + PFT-α-treated group (7.04 ± 1.52-fold, P < 0.05; and 4.42 ± 0.96, P < 0.05, respectively). These results indicate that TBI-induced HO-1 expression is dependent on p53. However, PFT-α itself induced neither HO-1 transcription nor its translation, and therefore was p53 independent. Since HO-1 mediates cellular responses to oxidative stress and autophagy, we further examined the effect of PFT-α on oxidative stress induced by the TBI. The expression of lipid peroxidation marker (4-HNE)-positive neurons in the striatum was assessed by immunofluorescence staining. Double immunofluorescence staining using an antibody against 4-HNE combined with a NeuN antibody showed that 4-HNE-positive neuron expression in the sham group was negligible (Fig. 7Aa–d). In the TBI-treated group, 4HNE-positive neurons had significantly increased (Fig. 6Ae–h), whereas this effect was reduced by PFT-α treatment (Fig. 7Ai-l). A quantitative analysis revealed that numbers of 4-HNE-positive neurons had significantly increased at 24 h in the TBI-treated group after injury (64.4% ± 14%; P < 0.001) compared to the sham group (0.7% ± 0.7%). PFT-α administration reduced the number of 4-HNE-positive neurons (27.8% ± 6.4%; P < 0.01) compared to the TBI-treated group (Fig. 7B). However, double immunofluorescent staining using anti-GFAP or anti-ED1 and anti-4-HNE antibodies showed that TBI-induced 4-HNE-positive cells were not expressed by GFAP-positive astrocytes or ED1-positive microglia (Fig. 7C). This result suggests that TBI-induced lipid peroxidation occurred mainly in neurons.

### Post-injury PFT-α treatment attenuates expression levels of autophagic markers in the striatum of rats with TBI

**Figure 8:**
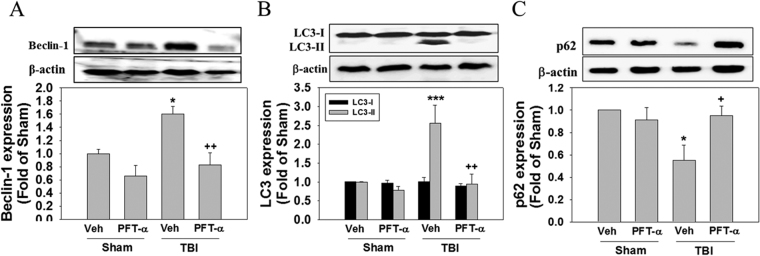
Post-injury administration of pifithrin (PFT)-α at 5 h after a traumatic brain injury (TBI) reduced levels of Beclin-1, light chain (LC)3-II and p62 expression in striatal tissues at 24 h. Levels of Beclin-1 (**A**), LC3 (**B**) and p62 (**C**) expression in striatal tissues from the Sham + Veh, Sham + PFT-α, TBI + Veh, and TBI + PFT-α groups were analyzed by Western blotting. Levels of Beclin-1, LC3I, LC3II and p62 expression were normalized to β-actin and then quantified. Data are expressed as the mean ± SD (*n* = 4 per group). *P < 0.05 and ***P < 0.001 vs. Sham + Veh group; ^+^P < 0.05 and ^++^P < 0.01 vs. TBI + Veh group.

Increasing evidence has demonstrated that perturbation of the autophagy pathway is associated with neuronal damage after TBI^41,42^. To investigate the effect of PFT-α on TBI-induced autophagy, expression levels of three autophagic markers (Beclin-1, LC3II, and p62) were assessed by Western blotting. Expression levels of Beclin-1 increased after injury compared to the Sham + Veh group (1.6 ± 0.11- vs.1.00 ± 0.07-fold, P < 0.05), whereas this effect was reduced by PFT-α treatment (0.86 ± 0.18- vs. 1.6 ± 0.11-fold, P < 0.01) (Fig. 8A). In the TBI + Veh group, the LC3II had significantly increased compared to the Sham + Veh group (2.55 ± 0.48- vs. 1.00 ± 0.01-fold, P < 0.001) (Fig. 8B). Post-injury treatment with PFT-α significantly reduced LC3II expression (0.93 ± 0.026- vs. 2.55 ± 0.48-fold, P < 0.01) (Fig. 8B). In addition, p62 protein levels were significantly lower in the TBI + Veh group compared to the Sham + Veh group (0.55 ± 0.13- vs. 1.00 ± 0.01-fold, P < 0.05) (Fig. 8C). Post-injury PFT-α treatment maintained the p62 protein level versus the TBI + Veh group (0.91 ± 0.1- vs. 0.55 ± 0.13-fold, P < 0.05, respectively; Fig. 8C). Levels of Beclin-1, LC3II and p62 expressions in the Sham + PFT-α group did not significantly differ from those observed in the Sham + Veh group. These results indicated that inhibition of p53 signaling by PFT-α is involved in TBI-induced autophagy formation in rats.

### Post-injury PFT-α treatment reduces TBI-induced neurodegeneration

**Figure 9:**
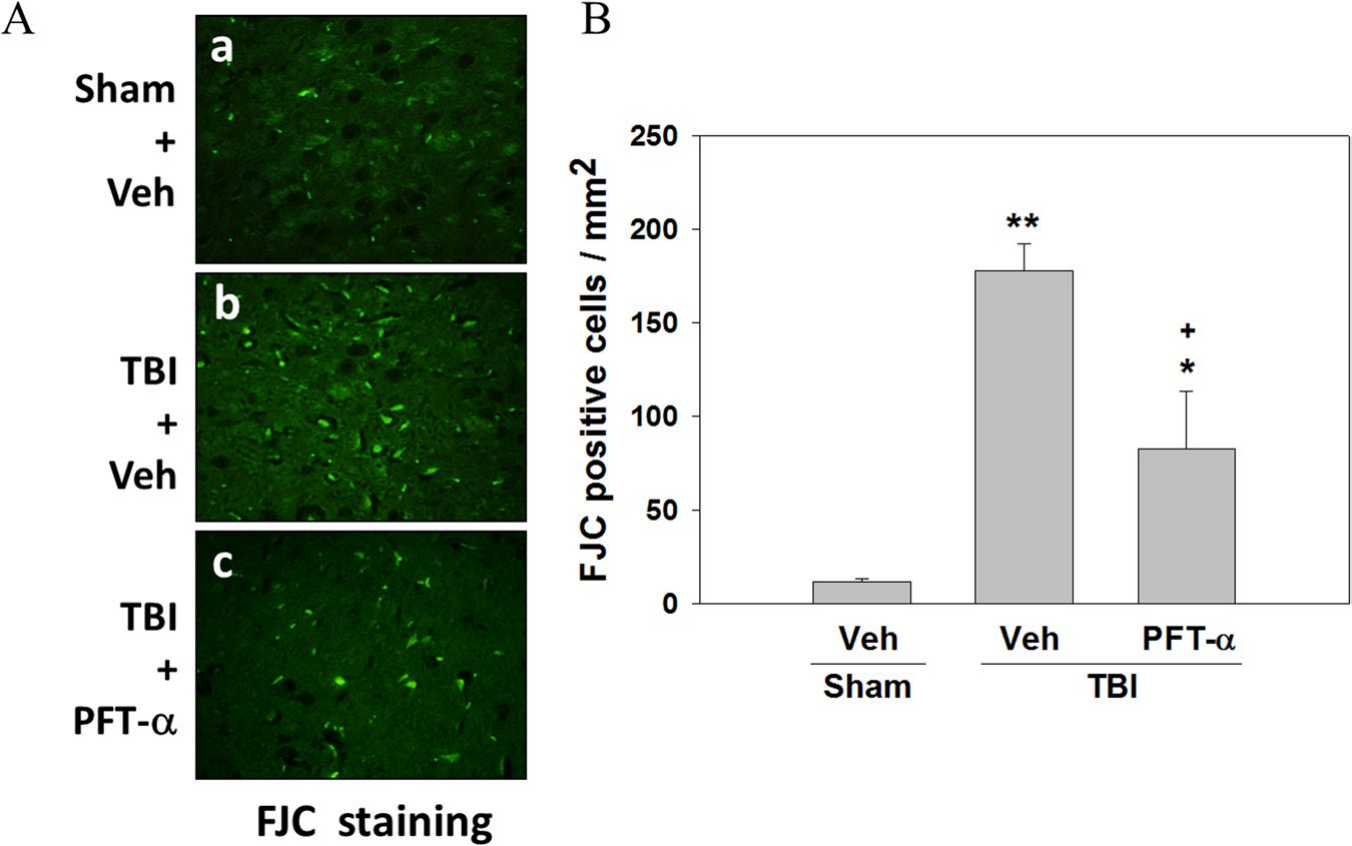
Post-injury administration of pifithrin (PFT)-α at 5 h after a traumatic brain injury (TBI) decreased Fluoro Jade C (FJC)-positive cells in striatal tissues at 24 h. (**A**) Photomicrographs of FJC-stained regions of interest in the Sham + Veh, lls. (**B**) Quantitative comparison of FJC-positive cells in the sham, TBI + Veh, and TBI + PFT-α groups at 24 h. The total number of FJC-positive cells is expressed as the mean number per field of view. Data are expressed as the mean ± SD (*n* = 4 per group). *P < 0.05 and **P < 0.01 vs. Sham + Veh group; ^+^P < 0.05 vs. TBI + Veh group.

Significant levels of apoptosis occur in the injured striatum after TBI. As illustrated in Fig. 9Ab, numerous FJC-positive cells were observed in the striatum at 24 h post-injury. A quantitative summary of FJC-positive cells observed in the Sham + Veh, TBI + Veh, and TBI + PFT-α groups is provided in Fig. 9B. Numbers of degenerating neurons were significantly elevated in the TBI + Veh group (177.9 ± 14.29/mm^2^, P < 0.01) compared to the sham group (12.167 ± 1.17/mm^2^). In comparison, the PFT-α-treated TBI group had a significantly lower number of degenerating neurons (82.73 ± 31.07/mm^2^, P < 0.05) compared to the TBI + Veh group.

### Post-injury PFT-α treatment attenuates expression of apoptotic-related genes and proteins in the striatum of rats with TBI

**Figure 10:**
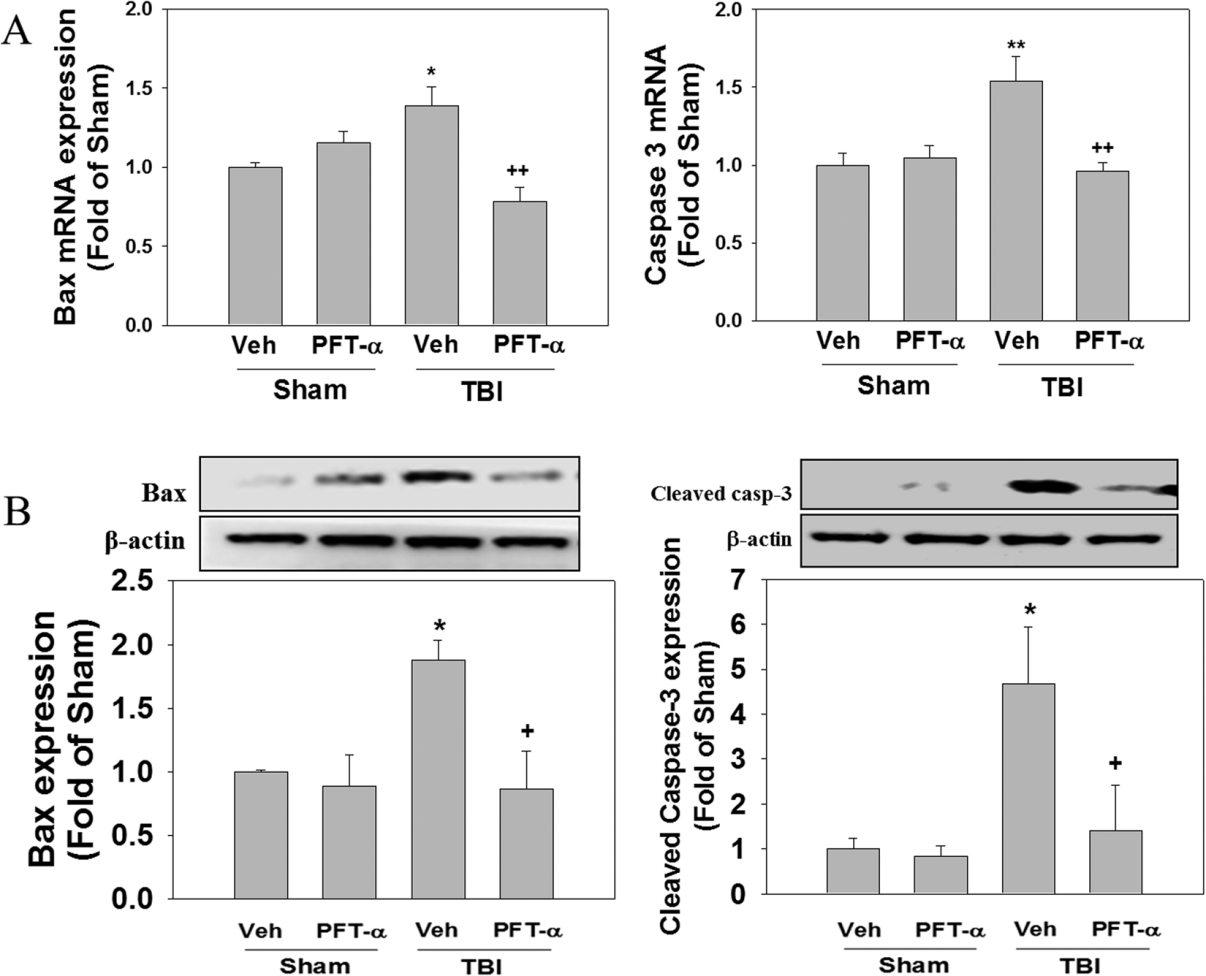
Post-injury administration of pifithrin (PFT)-α at 5 h after a traumatic brain injury (TBI) reduced both transcriptional and translational expression levels of Bax and cleaved caspase-3 in striatal tissues at 24 h. (**A**) Levels of Bax and caspase-3 mRNA expression in striatal tissues from the Sham + Veh, Sham + PFT-α, TBI + Veh, and TBI + PFT-α groups were analyzed by an RT-PCR. (**B**) Levels of Bax and cleaved caspase-3 protein expression in striatal tissues from each group were analyzed by Western blotting. Levels of mRNA and protein expression were normalized to β-actin and then quantified. Multiples of change are relative to that of the sham group. Data are expressed as the mean ± SD (*n* = 3 per group). *P < 0.05 and **P < 0.01 vs. Sham + Veh group; ^+^P < 0.05 and ^++^P < 0.01 vs. TBI + Veh group.

To investigate the effect of PFT-α on TBI-induced apoptosis, expression levels of Bax and caspase-3 mRNAs and proteins were respectively examined by an RT-PCR and Western blotting. Levels of Bax and caspase-3 mRNA expression in the Sham + PFT-α group did not significantly differ from these observed in the Sham + Veh group (Fig. 10A). Results showed that Bax and caspase-3 mRNA levels were significantly elevated in the TBI-treated group (1.49 ± 0.17-fold, P < 0.05 and 1.47 ± 0.21-fold, P < 0.01, respectively) compared to the sham-treated group (1. ± 0.11-fold and 1 ± 0.09-fold, respectively), whereas these effects were attenuated in the PFT-α-treated group (0.64 ± 0.05-fold, P < 0.001 and 0.61 ± 0.18, P < 0.05, respectively) (Fig. 10A). Levels of Bax and cleaved-caspase-3 proteins had significantly increased in the TBI-treated group (1.87 ± 0.15-fold, P < 0.05 and 4.68 ± 1.26-fold, P < 0.05, respectively; Fig. 10B), however both Bax and cleaved-caspase-3 expression were significantly reduced in the PFT-α treatment group (0.86 ± 0.29-fold, P < 0.05 and 1.41 ± 1.01-fold, P < 0.05, respectively). Levels of Bax and caspase-3 protein expression in the Sham + PFT-α group did not significantly differ from these observed in the Sham + Veh group. These results suggest that inhibition of p53 activation by PFT-α ameliorates striatal apoptotic cell death in rats following TBI.

### Post-injury PFT-α treatment attenuates neuronal apoptosis in the striatum of rats with TBI

**Figure 11:**
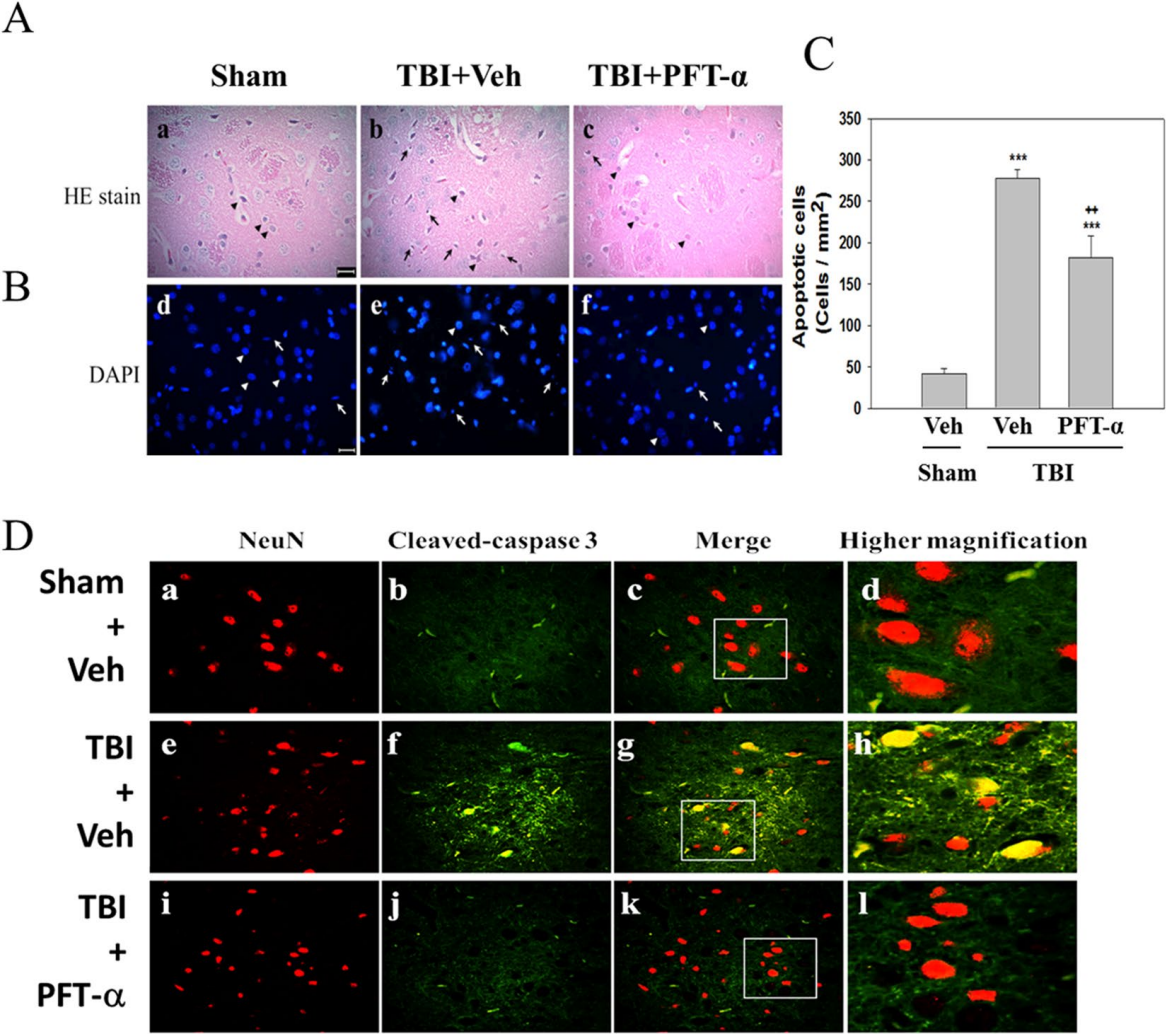
Post-injury administration of pifithrin (PFT)-α at 5 h after a traumatic brain injury (TBI) attenuated morphological abnormalities in the striatum of TBI rats. (**A**) Photomicrographs of H&E-stained sections of striatal tissues from the Sham + Veh (a), TBI + Veh (b), and TBI + PFT-α (c) groups. Arrows indicate cells in the striatum of TBI rats exhibiting cell shrinkage and nuclear pyknosis. (**B**) Representative photomicrographs of chromatin staining with DAPI showing changes in the morphology of nuclei (d,e,f). Sections were examined under a microscope after chromatin staining with DAPI. Arrowheads indicate normal nuclei observed in normal cells. Arrows indicate chromatin condensation, a reduced nuclear size, and nuclear fragmentation typically observed in apoptotic cells. Scale bar = 50 µm. (**C**) Quantitative comparison of apoptotic cells in each group. Data are expressed as the mean ± SD (*n* = 4 per group). ***P < 0.001 vs. Sham + Veh group; ^++^P < 0.01 vs. TBI + Veh group. (**D**) Double immunofluorescence staining of NeuN and cleaved caspase-3 (a marker for apoptotic cells) in striatal brain section. NeuN immunoreactivity is shown in red, and cleaved caspase-3 is shown in green, and colocalization of NeuN and cleaved caspase-3, indicating neuronal apoptosis, is shown in yellow. Scale bar = 50 µm.

H&E staining was performed to observe morphological abnormalities in the striatum after TBI. No significant abnormalities were observed in the sham group. In the TBI-treated group, a histological examination showed cell body shrinkage and nuclear pyknosis at 24 h after injury compared to the sham group, whereas this effect was attenuated by PFT-α treatment (Fig. 11A). This result was further confirmed by chromatin staining with DAPI. We found that nuclei of cells from the sham group were uniformly stained without condensation. However, nuclei from the TBI-treated group displayed morphological features that were typical of apoptosis, including a reduced nuclear size, high condensation, nuclear fragmentation, and some scattered apoptotic bodies. Post-injury treatment with PFT-α significantly ameliorated these nuclear abnormalities (Fig. 11B). A quantitative analysis revealed that numbers of apoptotic cells had significantly increased at 24 h in the TBI-treated group after injury (277.7 ± 11.1 cells/mm^2^; P < 0.001) compared to the sham group (41.5 ± 6.5 cells/mm^2^). PFT-α administration reduced the number of apoptotic cells (182 ± 26.5 cells/mm^2^; P < 0.01) compared to the TBI-treated group (Fig. 11C). We further confirmed the effect of PFT-α on neuronal apoptotic cell death in striatal tissues at 24 h after a TBI using double immunofluorescence staining. Double immunofluorescence staining using an antibody against cleaved caspase-3 combined with a NeuN antibody showed that neurons positive for cleaved caspase-3 expression in the sham group were negligible compared to those observed in the TBI-treated group (Fig. 11Da–h, respectively). Post-injury treatment with PFT-α at 5 h reduced the number of cleaved caspase3-positive neurons (Fig. 11Di–l). These findings underscore that striatal damage plays a critical role in the pathological process of TBIs.

## Discussion

Although animal studies demonstrated that TBIs cause cortical and hippocampal neuronal loss^42–44^, only a few studies have focused on striatal damage caused by TBI^3,45^. The striatum is one of the major entries into the basal ganglia. Striatal neurons can receive glutamate inputs from the cortex and dopamine (DA) innervation from the midbrain. Striatal dysfunction is associated with deficits in learning, memory, and executive function following TBI^46^. Multiple lines of evidence imply that DA-targeted therapies represent an important clinical option for treating persistent cognitive dysfunction after TBI^3,45,46^. Those results underscore that the striatum plays an important role in neurological function. The present work was performed in an effort to determine the effects of PFT-α, a p53 inhibitor, on TBI-induced striatal damage. We report a possible molecular mechanism of PFT-α against TBI damage in the striatum through amelioration of neurological functional deficits, attenuating neuroinflammation, oxidative stress, autophagy, and apoptosis following TBI.

The transcriptional factor, p53, plays various distinct roles in response to cell stresses, whereas inappropriate p53 activation is involved in stress-induced apoptotic signaling pathways in neurological diseases and brain injuries^13,47–50^. Animal studies demonstrated that levels of p53 mRNA and protein were increased in the cortex and hippocampus after TBI^48,49^. Our previous study demonstrated that post-injury PFT-α administration reduced p53 mRNA and p53-positive neurons in the cortex and hippocampus^44,51^. Recent studies mainly focused on the role of p53 in neurons, and it is known that inhibition of p53 significantly reduced neuronal cell death^15,16,44^. However, it was reported that p53 is also inducible in astrocytes and microglia under pathological conditions^52,53^. For example, 1 or 3 days following ischemic injury, p53 was not obviously detectable in reactive astrocytes in the forebrain. However, p53 expression in reactive astrocytes was clearly detectable in affected cortical regions (e.g., the forelimb area, hindlimb area, and parietal cortex) at 7 days following ischemic injury^54^. It was also demonstrated that p53 was detectable in neurons, activated microglia, and astrocytes at 24 h after hypoxia-ischemia (HI) injury on postnatal day 7 in rat pups. Expressions of microglia marker proteins are dependant on specific differentiation states in CNS diseases or disease models^55,56^, indicating that microglia are capable of heterogeneous functional differentiation responses to disease-relevant stimuli. It was reported that p53 mainly influences M1-like microglial behavior, supporting the adoption of a proinflammatory phenotype^57,58^, but p53 activation is excluded from the population of microglia labeled by CD163^57^, a marker of macrophages that have adopted anti-inflammatory or tissue repair phenotypes (M2-like microglia). In the present study, we observed that p53 mRNA significantly increased in the striatum, whereas p53 expression was predominantly distributed in neurons but not in microglia or astrocytes in the striatum at 24 h after the TBI. The absence of p53 expression in astrocytes and microglia is likely because the expression of p53 in activated microglia and astrocytes possibly depends upon the specific disease model employed, the timing of glial activation, the microglial phenotype, and specific brain regions examined. Moreover, we found that post-injury treatment with PFT-α reduced p53 expression and improved sensory-motor function (as revealed by a tactile adhesive removal test) and neurological deficit severity (as revealed by the mNSS test), indicating that p53 may participate in striatal pathophysiological processes of TBIs. Since there was no difference between Sham + Veh group and Sham + PFT-α group, the slight difference between TBI + PFT-α group and Sham + Veh may be due to a multiple effect of PFT-α on TBI-induced p53 mRNA transcription including direct effect and indirect effect on other TBI-activated pathways that influence p53 transcription. Our speculation may need further investigation.

The underlying mechanisms of TBIs are complicated. Neuroinflammation is one of the crucial factors among the pathophysiological manifestations of TBIs^59,60^. The presence of a number of markers of neuroinflammation is evident in insult regions, such as elevated production of proinflammatory cytokines and ROS by activated glial cells^60–62^. Emerging evidence suggests that p53 is primarily involved in cancer biology and also participates in various novel functions in the brain, such as modulation of glial function and mediation of the pathophysiological process of neurodegenerative disease^39,63^. For example, induction of microglial p53 expression by neurotoxic activators (e.g., amyloid beta peptide and lipopolysaccharide) caused cerebellar granule neuronal toxicity, whereas inhibition of p53 by PFT-α reduced neurotoxicity via modulation of induced inducible nitric oxide synthase expression and TNF-α secretion^63^. p53 and NF-κB were shown to engage in functional cross-talk and participate in modulation of inflammatory responses^64,65^, indicating that p53 plays a critical role in innate immune function. In this study, we found that striatal astrocytes displayed a hypertrophic size and microglia displayed an amoeboid morphology (both characteristics of activated glia) which were accompanied by TNF-α, IL-1β, and IL-6 cytokine mRNA expression and protein production after TBI. However, morphological features of activated glia and expression of cytokines were attenuated by PFT-α, indicating that TBI-induced glial activation and neuroinflammation are possibly controlled, at least in part, via p53; however, the exact cellular and molecular mechanisms remain to evaluated in the future.

It is now generally accepted that neuronal cell death due to oxidative stress is a common characteristic of TBIs^66–68^. The degree of oxidative stress in different brain regions (e.g., the cortex, hippocampus, and striatum) is dependent on the TBI intensity^36^. In line with our observations, there are a number of studies showing that treatment with antioxidant N-acetyl cysteine (NAC) or glutathione reduced the damage produced by a TBI in preclinical models^69,70^. The p53 tumor suppressor protein was proposed as a key mediator of stress responses because it plays an essential role in neuronal cell death^66–68^. It was demonstrated that p53 induces cell death by multiple molecular pathways involving regulation of antioxidant, pro-oxidant, proapoptotic, and autophagic gene expression^71^. The brain is highly sensitive to lipid peroxidation (LP) due to its high contents of polyunsaturated fatty acids and high levels of iron^72^. The most notable end products of LP, 4-hydroxynonenal (4-HNE), can bind to proteins, modifying their structures and functions, which in turn causes neuronal damage^66,73^. Consistent with that study, levels of malondialdehyde (MDA), the end product of LP, increased after TBI; however, treatment with the DA D2 receptor agonist, bromocriptine, attenuated levels of MDA production and improved histological and cognitive outcomes^74^. Those results indicate that oxidative stress plays an important role in TBIs. HO-1, which is associated with the endogenous defense response, represents an important cellular cytoprotective mechanism against oxidative damage by degrading toxic heme into free iron, carbon monoxide, and biliverdin^75,76^. It was proposed that brain redox homeostasis (e.g., HO-1) can be regulated by the interplay between superoxide dismutase 2 and p53^77^. In the present study, we found that HO-1 expression was upregulated after a TBI, whereas post-injury treatment with the p53 inhibitor, PFT-α, enhanced the upregulation of HO-1 mRNA and protein increases, compared to the TBI group. Since HO-1 mediates cellular responses to oxidative stress, we further examined the effect of PFT-α on oxidative stress induced by the TBI. We found that the number of 4-HNE-positive cells significantly increased in the rat striatum after the TBI, whereas this effect was reduced by PFT-α. Our results showed that TBI-induced lipid oxidation mainly occurs in neurons but not in astrocytes or microglia. The possible explanation for this observation is that neurons, which contain lower levels of glutathione compared to glia, are more vulnerable to oxidative damage^78^. In addition, the presence of a number of markers of neuroinflammation is evident in injured regions, such as elevated production of proinflammatory cytokines, and ROS by activated glial cells^79,80^. Sustained production of these factors contributes to neuronal oxidative damage^81–83^. However, whether glial cell activation in the brain is beneficial or harmful remains unclear. Most likely glial activation may be both beneficial and harmful, depending upon the degree and longevity of the activity. Hence, expressions and releases of neurotrophic or harmful substances in response to neuronal injuries are likely determinants of neuronal viability. Our findings suggest that PFT-α attenuates TBI-induced oxidative stress possibly via inducing HO-1 expression and attenuating the number of 4-HNE-positive cells. Collectively, these results highlight the important role of p53 in counteracting TBI-induced oxidative stress. However, questions remain as to how PFT-α can reduce oxidative damage after TBI.

Since HO-1 also mediates cellular responses to autophagy, we also examined the role of p53 in TBI-induced autophagy. Emerging evidence reported that oxidative stress is essential for induction of autophagy^24,84^. Increased autophagy was demonstrated to be involved in animal models of TBI^18,42,85^. Basal expression of autophagy is a homeostatic process of recycling dysfunctional macromolecules and organelles in physiological conditions^22,23^. However, under certain pathological circumstances (e.g., oxidative stress), autophagy increases and is perhaps involved in cytoprotection or cell death. Beclin-1, a marker of autophagic activation, is a key protein involved in regulating autophagy^86^. LC3-II is another autophagic marker. Conversion of LC3-I to LC3-II via the addition of phosphatidylethanolamine is essential for the formation of autophagosomes, and expression of the LC3-II protein can reflect autophagic activity^87,88^. The sequestosome 1 (SQSTM1)/A170 protein, also known as the p62 protein, was suggested to be correlated with ubiquitinated proteins and directly binds to LC3, which may regulate the selective autophagic clearance of the p62 substrate^89^. In the present study, we found that post-injury PFT-α treatment significantly decreased TBI-induced elevation of Beclin and LC3II, and maintained p62 levels in the striatum, suggesting a decreased ability of autophagy to degrade p62. A possible mechanism may be supported by this study which showed that p53 can inhibit mTOR, a suppressor of autophagy^20^, indicating that p53 has an important role in autophagy regulation after TBI. In accordance with these biochemical changes, our histologic evaluation revealed that PFT-α also significantly reduced FJC-positive cells (a marker of degenerating neurons) and conferred neuroprotection. It is known that oxidative stress-induced DNA damage can overwhelm repair systems, and activation of p53 cascades may initiate p53-mediated apoptosis^66^. Proapoptotic-related proteins, such as Bax and caspase-3, can be induced by TBI^90^. Upregulation of p53 and a decrease in the ratio of Bcl-2 (an antiapoptotic protein) to Bax (a proapoptotic protein) accompanied neuronal apoptosis in a closed-head injury rat model^38^. In this study, PFT-α administration post-injury reduced mRNA and protein levels of Bax and caspase-3 in striatal tissues after TBI. These results were further confirmed by fewer apoptotic nuclei in PFT-α-treated than vehicle-treated TBI animals using DAPI staining. Taken together, pharmacological inhibition of p53 by PFT-α attenuated TBI-induced autophagy and neuronal apoptotic cell death, underscoring the importance of p53 activation in the pathophysiology of TBIs.

Whereas most published articles on PFT-α are consistent with its p53 inhibitory activity, it is known that PFT-α analogs also possess some p53-independent effects, particularly at high concentrations. It was shown that PFT-α is a potent agonist of the aryl hydrocarbon receptor (AhR), and that this activity of PFT-α is not related to its p53-inhibitory properties^91^. In addition, PFT-α can directly inhibit the catalytic activities of recombinant isoforms of the human cytochrome P450 superfamily of enzymes, CYP1A1, CYP1A2, and CYP1B1. Although this has yet to be evaluated *in vivo*, this observation provides a plausible explanation for the protective actions of PFT-α against chemical carcinogens^92^. Finally, at least in some cell systems, PFT-α is capable of suppressing the heat shock response and inhibiting glucocorticoid receptor signaling^93,94^. These examples show that the actions of PFT-α depend to some degree on the cell type evaluated, particularly whether of neuronal or non-neuronal origin. It is also likely such distal effect of injury is dependent on injury intensity so that it would be important to assess the time course of striatal p53 upregulation, immune response, neurodegeneration, oxidative stress, autophagy apoptosis, activation of glial cells and neuroinflammation after TBI for the delineation of primary and secondary injury and injury progression. However, these events are all maximized at specific time points after the TBI. We chose to measure these parameters at their maximum values based on our previous studies to demonstrate the efficacy of PFT-α.

In conclusion, results of the present study suggest that mechanisms underlying the neuroprotective effects of PFT-α may act in part through amelioration of neurological functional deficits, attenuating neuroinflammation, oxidative stress, autophagy, and apoptosis in the striatum following an experimental TBI in rats. Although such striatal changes could be indirect effect due to less damages in cortex or hippocampus but not due to direct regulation of immune response, astrocytes activation, HO-1 upregulation following our TBI protocol^44,51^, such a possibility will require further investigation. In any event, inhibition of p53-mediated pathophysiological processes by PFT-α can possibly be developed into a novel therapeutic strategy for TBIs. Hence, further evaluation and optimization of PFT-α for potential applications in clinical TBIs are required.

## Methods

### Animal model of TBI using a controlled cortical impactor (CCI)

All animals were treated in accordance with international guidelines for animal research, and the study design was approved by the animal ethics committee of Taipei Medical University. Animals were housed in groups in a temperature- (21~25 °C) and humidity (45~50%) controlled room with a 12-h light/dark cycle and ad libitum access to pellet chow and water. A CCI was used to produce TBI in rats as previously described^44,95^. Male Sprague-Dawley rats (250~300 g in body weight) were randomized into four groups. Sham animals received anesthesia and a craniotomy but no TBI, then received either a Veh (10% DMSO in saline, iv.) or a PFT-α (a p53 inhibitor) injection (2 mg/kg, iv.) at 5 h after the craniotomy, and were respectively designated the Sham + Veh or Sham + PFT-α group. Another two groups of animals were anesthetized and placed in a stereotaxic frame and subjected to a TBI. Injury was made using a CCI instrument with a rounded metal tip (5 mm in diameter) as we previously described^44,51,96^. Briefly, a craniotomy was performed over the left parietal cortex, centered on the coronal suture and 3.5 mm lateral to the sagittal suture. A velocity of 4 m/s and a deformation depth of 2 mm below the dura were used. TBI animals also received either the Veh or PFT-α at 5 h post-injury and were designated the TBI + Veh or TBI + PFT-α group, respectively. The body temperature was monitored throughout the surgery with a rectal probe; it was maintained at 37 °C using a heating pad. Rats were placed in a heated cage to maintain their body temperature while recovering from anesthesia. No temperature changes were seen after PFT-α administration in normal or sham animals. Behavioral and histological analyses were carried out by an experimenter blinded to the treatment conditions. All animals were sacrificed at 24 h after the craniotomy. Figure 1 depicts the grouping of sham and TBI animals receiving Veh or PFT-α for morphological evaluations or biochemical measurements of messenger (m)RNA and protein levels. The “striatal region” we isolated and dissected for biochemical measurements included both the dorsal and ventral striatum. However, all the immunostaining images were taken from the dorsal striatum. For morphological studies, the brain of each animal was sectioned (10 μm). Sections were selected for HE, FJC, DAPI, and immunofluorescence staining using standard methods. Our selected treatment time of 5 h and dosage were based on our prior study indicating that the secondary phase of neuronal injury following TBI can be attenuated if appropriate treatment is initiated within this time window^44^.

### Behavioral evaluation of neurological outcomes

Behavioral testing was performed before the TBI and at 24 h after the TBI. The evaluation consisted of a beam walk test, an elevated body swing test (EBST), a tactile adhesive removal test, and a modified neurological severity score (mNSS) assessment. All were performed by an observer blinded to the experimental groups. These procedures were performed as previously described^44,97,98^, with some modifications. Briefly, (1) the beam walk test was used to assess the fine motor coordination and balance^97^. Five trials were recorded 1 h before the TBI (baseline) and at 24 h after the TBI. Mean values of the latency were then quantified. (2) Body asymmetry was quantitatively analyzed with the use of the EBST. As the TBI was performed on the left side, resulting weakness (hemiparesis) developed on the right side. The frequency of left/right swings was scored across 20 consecutive trials and expressed as a percentage [calculated by: (number of right-biased swings/the total number of swings) × 100%]. An uninjured animal showed an equal frequency to swing to either on the left or right side. (3) A tactile removal test was used to evaluate somatosensory function. Two small adhesive-backed stickers (each 113.1 mm^2^) were used as bilateral tactile stimuli that were placed on the distal-radial region of the wrist of each forelimb^98^. Rats were pre-trained daily for 3 days before the TBI. The time required for the rat to remove the sticker from the forelimb was recorded in five trials at 24 h after the TBI. (4) To compare the neurological deficit severity in TBI rats, an mNSS which is a composite of motor, sensory, reflex, and balance tests was performed^98^. One point was scored for the inability to perform the test or for the lack of a tested reflex; thus, the higher the score, the more severe the injury. Neurological function was graded on a scale of 0~18 (normal score, 0; maximal deficit score, 18).

### Measurement of cytokines

Concentrations of cytokines (TNF-α, IL-1β, and IL-6) were measurement by enzyme-linked immunosorbent assay (ELISA) kits (BioSource International, Camarillo, CA, USA) according to the manufacturer’s protocol.

### Western blot analysis

Under deep anesthesia (sodium pentobarbital i.p.; 50 mg/kg), rats were euthanized at 24 h. The striatal region of the brain was rapidly isolated and homogenized, and total proteins were extracted using protein extraction buffer (Mammalian Cell-PE LBTM, Geno Technology, USA) containing protease and phosphatase inhibitors (Complete Mini, Roche Diagnostics, Indianapolis, IN, USA). Lysed tissues were centrifuged at 12,500 rpm for 15 min (at 4 °C), and the supernatant was collected for storage at −80 °C or to perform a Western blot analysis. Proteins were separated by gel electrophoresis, and then electroblotted onto polyvinylidene difluoride (PVDF) membranes (PerkinElmer Life Sciences, USA). Membranes were blocked with 5% non-fat milk, and then incubated overnight at 4 °C with the indicated antibodies, including heme oxygenase (HO)-1, Bax, cleaved caspase-3, Beclin-1, LC3-I/II, and p62 (1:10^3^ dilution) followed by an appropriate secondary antibody (1:2 × 10^4^ dilution) for 1 h at room temperature. Signals were visualized using enhanced chemiluminescent (ECL) detection reagents (PerkinElmer Life Sciences). The membrane was then stripped and reprobed with an antibody specific for β-actin (1:10^4^ dilution) to ensure the accuracy of each loading. Protein expression was quantified by a BioImaging System (Level Biotechnology).

### Real-time reverse-transcription polymerase chain reaction (RT-PCR) assay

Total RNA was extracted from striatal tissues using the TRIzol® reagent (Invitrogen Life Technologies, Paisley, Scotland). Total RNA (3 µg) was reverse-transcribed into complimentary (c)DNA using the Rever Tra Ace-α First-strand cDNA Synthesis Kit (Toyobo Life Science, Japan). The resulting cDNA was incubated with a Rotor-Gene SYBR Green kit (Applied Biosystems, Foster City, CA, USA) and primers. Primers sequence used in the RT-PCR are shown in Table 1. For the semiquantitative analysis, we performed 40 amplification cycles (denaturation at 95 °C for 5 s and annealing at 60 °C for 10 s) on a Rotor-Gene Q PCR Detection System (Qiagen Biosystems, CA, USA). Melting curve and sequencing data were used to confirm the specificity of the polymerase chain reaction (PCR) products. Levels of target genes were normalized to those of β-actin and were then expressed as values relative to the control using the comparative threshold cycle (Ct) method.

**Table 1:** Primer secquences.

Gene of interest	Forward Primer (5′-3′)	Reverse Primer (5′-3′)
TNF-α	CTCTTCTCA TTCCCGCTCGTG	GGAACTTCTCCTCCTTGTTGGG
IL-1β	GTTTGAGTCTGCACAGTTCCC	CAACTATGTCCCGACCATTGC
IL-6	TTCTTGGGACTGATGTTGTTGAC	AATTAAGCCTCCGACTTGTGAAG
HO-1	GAGATAGAGCGAAACAAGCAGAAC	GTCCTGTACCGGAAGACCATAC
p53	CCATGGCCATCTACAAGAAGT	TGTCGTCCAGATACTCAGCATA
Bax	GTGAGCGGCTGCTTGTCT	GTGGGGGTCCCGAAGTAG
Caspase 3	AATTCAAGGGACGGGTCATG	GCTTGTGCGCGTACAGTTTC
β-actin	GACCCAGATCATGTTTGAGACCTTC	GGTGACCGTAACACTACCTGAG

### Fluoro jade C (FJC) staining

FJC, a derivative of polyanionic fluorescein, selectively binds to degenerating neurons. We used an FJC ready-to-dilute staining kit (TR-100-FJ, Biosensis) to identify degenerating neuronal cells according to the manufacturer’s protocol with some modifications. Brain sections from the different treatment groups were deparaffinized, rehydrated, and incubated in distilled water for 2 min. Brain sections were incubated in a solution of potassium permanganate (1:15) for 10 min, rinsed in distilled water for 2 min, and then incubated in the FJC solution (1:25) for 30 min. After incubation, brain slides were washed and mounted on coverslips with Vecta-shield mounting medium (Vector Laboratories, Burlingame, CA, USA). All sections were observed and photographed using a fluorescent inverted microscope (IX70, Olympus, Japan). Numbers of FJC-positive cells were counted in three randomly selected fields per slide by means of SPOT image analysis software (Diagnostic Instruments). Numbers of FJC-positive cells observed on the slides from different treatment groups were counted and used to generate a mean number per treatment group.

### Double immunofluorescence staining

Coronal sections (10 µm) were obtained from the anterior area of the left hemisphere. All sections were dried, rehydrated in phosphate-buffered saline (PBS), and rinsed in PBS. Sections were blocked for 60 min in 5% bovine serum albumin (BSA) (PBS containing 5% BSA and 0.2% Triton X-100; Sigma, St. Louis, MO, USA) and incubated with the appropriate primary antibodies, either an anti-NeuN antibody (a neuronal marker; mouse monoclonal anti-NeuN from Millipore, USA; rabbit polyclonal anti-NeuN from Cell Signaling, USA; 1:500), anti-GFAP antibody (an astrocyte marker; mouse monoclonal anti-GFAP from Abcam, USA; rabbit polyclonal anti-GFAP from Cell Signaling, USA; 1:1000), mouse anti-ED1 (a microglial marker, Serotec), rabbit anti-Iba1 (a microglial marker, GeneTex), mouse anti-p53 antibody (Abcam, USA; 1:300), rabbit anti-4-HNE antibody (Abcam; 1:300), or rabbit anti-cleaved caspase-3 (Cell Signaling, USA; 1:300) at 4 °C overnight and with a secondary antibody (Alexa Fluo® 488 goat anti-rabbit/anti-mouse immunoglobulin G (IgG) or Alexa Fluo® 594 goat anti-rabbit/anti-mouse IgG; Jackson ImmunoResearch, West Grove, PA, USA; 1:250) for 1 h at room temperature. Sections were mounted with mounting medium H-1000 (Vector Laboratories). Immunofluorescence images were viewed using an inverted Olympus IX 70 microscope equipped with a cooled CCD camera and SPOT advanced software (Diagnostic Instruments, Sterling Heights, MI, USA). Images of NeuN-p53-positive cells or NeuN-4-HNE-positive cells in the sham, TBI + Veh, and TBI + PFT-α groups (in three randomly selected fields within the calibrated area) were captured in a personal computer as digital micrographs and counted.

### Hematoxylin and eosin (H&E) staining

Histological and morphological abnormalities in the striatum of the sham, TBI + Veh, and TBI + PFT-α groups were examined with an H&E staining kit (GeneCopoeia^TM^, USA) according to the manufacturer’s protocol.

### Morphological analysis of apoptotic nuclei

To visualize apoptotic nuclei, sections were fixed in 4% paraformaldehyde (PFA) and stained with the fluorescent DNA-binding dye, 4’,6-diamidino-2-phenylindole (DAPI) (5 µg/ml, Sigma-Aldrich, St. Louis, MO, USA) for 1 min. After incubation, brain slides were washed and mounted on coverslips with Vecta-shield mounting medium (Vector Laboratories). Images of apoptotic cells in the sham, TBI + Veh, and TBI + PFT-α groups (in three randomly selected fields within the calibrated area) were captured in a personal computer as digital micrographs and counted.

### Statistical analysis

All data are presented as the mean ± standard error of the mean (SEM). Statistical significance was assessed by a one-way analysis of variance (ANOVA) followed by Newman-Keuls’ test using the SigmaStat program (Jandel Scientific, San Rafael, CA, USA). Significance was set at P < 0.05.
